# Transcranial Magnetic Stimulation–Induced Heart-Brain Coupling: Implications for Site Selection and Frontal Thresholding—Preliminary Findings

**DOI:** 10.1016/j.bpsgos.2023.01.003

**Published:** 2023-01-24

**Authors:** Eva Dijkstra, Hanneke van Dijk, Fidel Vila-Rodriguez, Lauren Zwienenberg, Renée Rouwhorst, John P. Coetzee, Daniel M. Blumberger, Jonathan Downar, Nolan Williams, Alexander T. Sack, Martijn Arns

**Affiliations:** a Heart & Brain Group, Brainclinics Foundation, Nijmegen, the Netherlands; b Department of Cognitive Neuroscience, Faculty of Psychology and Neuroscience, Maastricht University, Maastricht, the Netherlands; c Neurowave, Amsterdam, the Netherlands; d Department of Psychiatry, University of British Columbia, Vancouver, British Columbia, Canada; e Synaeda Psycho Medisch Centrum, Leeuwarden, the Netherlands; f Neurocare group Netherlands, The Hague, the Netherlands; g Department Of Psychiatry and Behavioral Sciences, Stanford University, Palo Alto, California; h Department of Psychiatry, University of Toronto, Toronto, Ontario, Canada; i Temerty Centre for Therapeutic Brain Intervention, Centre for Addiction and Mental Health, Toronto, Ontario, Canada

**Keywords:** Dash rTMS, Frontal-vagal network, Heart-brain coupling, Heart rate, iTBS, NCG-TMS

## Abstract

**Background:**

Neurocardiac-guided transcranial magnetic stimulation (TMS) uses repetitive TMS (rTMS)–induced heart rate deceleration to confirm activation of the frontal-vagal pathway. Here, we test a novel neurocardiac-guided TMS method that utilizes heart-brain coupling (HBC) to quantify rTMS-induced entrainment of the interbeat interval as a function of TMS cycle time. Because prior neurocardiac-guided TMS studies indicated no association between motor and frontal excitability threshold, we also introduce the approach of using HBC to establish individualized frontal excitability thresholds for optimally dosing frontal TMS.

**Methods:**

In studies 1A and 1B, we validated intermittent theta burst stimulation (iTBS)–induced HBC (2 seconds iTBS on; 8 seconds off: HBC = 0.1 Hz) in 15 (1A) and 22 (1B) patients with major depressive disorder from 2 double-blind placebo-controlled studies. In study 2, HBC was measured in 10 healthy subjects during the 10-Hz “Dash” protocol (5 seconds 10-Hz on; 11 seconds off: HBC = 0.0625 Hz) applied with 15 increasing intensities to 4 evidence-based TMS locations.

**Results:**

Using blinded electrocardiogram-based HBC analysis, we successfully identified sham from real iTBS sessions (accuracy: study 1A = 83%, study 1B = 89.5%) and found a significantly stronger HBC at 0.1 Hz in active compared with sham iTBS (*d* = 1.37) (study 1A). In study 2, clear dose-dependent entrainment (*p* = .002) was observed at 0.0625 Hz in a site-specific manner.

**Conclusions:**

We demonstrated rTMS-induced HBC as a function of TMS cycle time for 2 commonly used clinical protocols (iTBS and 10-Hz Dash). These preliminary results supported individual site specificity and dose-response effects, indicating that this is a potentially valuable method for clinical rTMS site stratification and frontal thresholding. Further research should control for TMS side effects, such as pain of stimulation, to confirm these findings.

Repetitive transcranial magnetic stimulation (rTMS) is a noninvasive neuromodulation technique which is increasingly used as an intervention for the treatment of refractory major depressive disorder (MDD) (1). rTMS for MDD is often targeted to the dorsolateral prefrontal cortex (DLPFC), and clinical response is thought to be mediated by network connectivity between the DLPFC and the subgenual anterior cingulate cortex (2). There is great potential to optimize rTMS parameters and move toward an individualized approach to rTMS therapy, including optimizing target engagement and parameters such as rTMS pattern, intensity, and frequency of stimulation (3,4).

It is well known that MDD is associated with an increased risk of cardiovascular disease, and heart rate (HR) is often dysregulated in MDD, quantified by a higher HR and lower HR variability (5). Relatedly, we recently proposed the frontal-vagal network theory for MDD, stating that major hubs such as the DLPFC, subgenual anterior cingulate cortex, and vagus nerve share overlap with the networks involved in autonomic control as well as in MDD (5). The neuroanatomical framework of the heart-brain connection in MDD is provided in (5). Based on this theory, a new target engagement method for treatment with rTMS was recently proposed by Iseger *et al.* (6), called neurocardiac-guided TMS (NCG-TMS). In NCG-TMS, HR deceleration in response to 10-Hz rTMS or intermittent theta burst stimulation (iTBS) is used to confirm activation of the frontal-vagal network in a site-specific manner, with HR deceleration at sites often used in TMS (e.g., F3, FC3) and HR acceleration in control sites such as the motor (C3, C4) or parietal (Pz) cortex (6).

This finding has now been replicated in healthy control subjects (5, 6, 7, 8) and in patients with MDD (9) and therefore holds promise as a possible biomarker of response and target engagement method allowing for determination of the best prefrontal rTMS target in MDD treatment (9). This is supported by preliminary results where HR deceleration at the first rTMS session was associated with clinical response after treatment (10). As an intermediate step, we recently proposed that NCG-TMS could be used as an enhanced rTMS stratification technique to select between one of the two evidence-based DLPFC sites: the Beam-cluster (Beam F3 and F4) and 5-cm rule (5CM) method (9).

Importantly, iTBS showed stronger effects on HR relative to standard 10-Hz rTMS (iTBS: 8.4 vs. 10 Hz: 1.9 beats per minute deceleration) (8,10), but it also resulted in more side effects, such as light-headedness, emotional reactions, and painfulness. For this reason, we set out to improve the current 10-Hz NCG-TMS method. Recently, the Food and Drug Administration approved the 10-Hz Dash protocol (11), where the intertrain interval (ITI) was shortened to 11 seconds, allowing for more rapid delivery of the stimulation trains during the session.

Given that the effects of TMS-induced HR deceleration are nearly immediate, because the vagus nerve is myelinated (whereas the sympathetic inputs to the heart are unmyelinated) [for review, see (5)], we hypothesized that specific TMS protocol parameters could induce entrainment of the cardiac rhythm as a function of TMS cycle time (Figure 1), as described in Thut *et al.* (12) for rTMS and electroencephalogram alpha oscillations. If entrained, the 2-second (iTBS) or 5-second (10-Hz) stimulation would decelerate HR during and immediately after each train, followed by a recovery of the HR during the ITI. Over the session with repeated trains, an entrainment of the interbeat interval at a specific frequency could be observed. The heart-brain directionality of TMS would thus be top-down from the brain on the HR (13). This hypothesis is visualized in Figure 1 for the 10-Hz Dash protocol (top) and iTBS (bottom). This novel heart-brain coupling (HBC) marker is proposed because HBC is independent of baseline HR state and other influences on HR, such as respiratory sinus arrhythmia, making it a more reliable and stable biomarker. Furthermore, respiratory sinus arrhythmia is generally faster (0.15–0.4 Hz) than these TMS cycle times (13).

**Figure 1:**
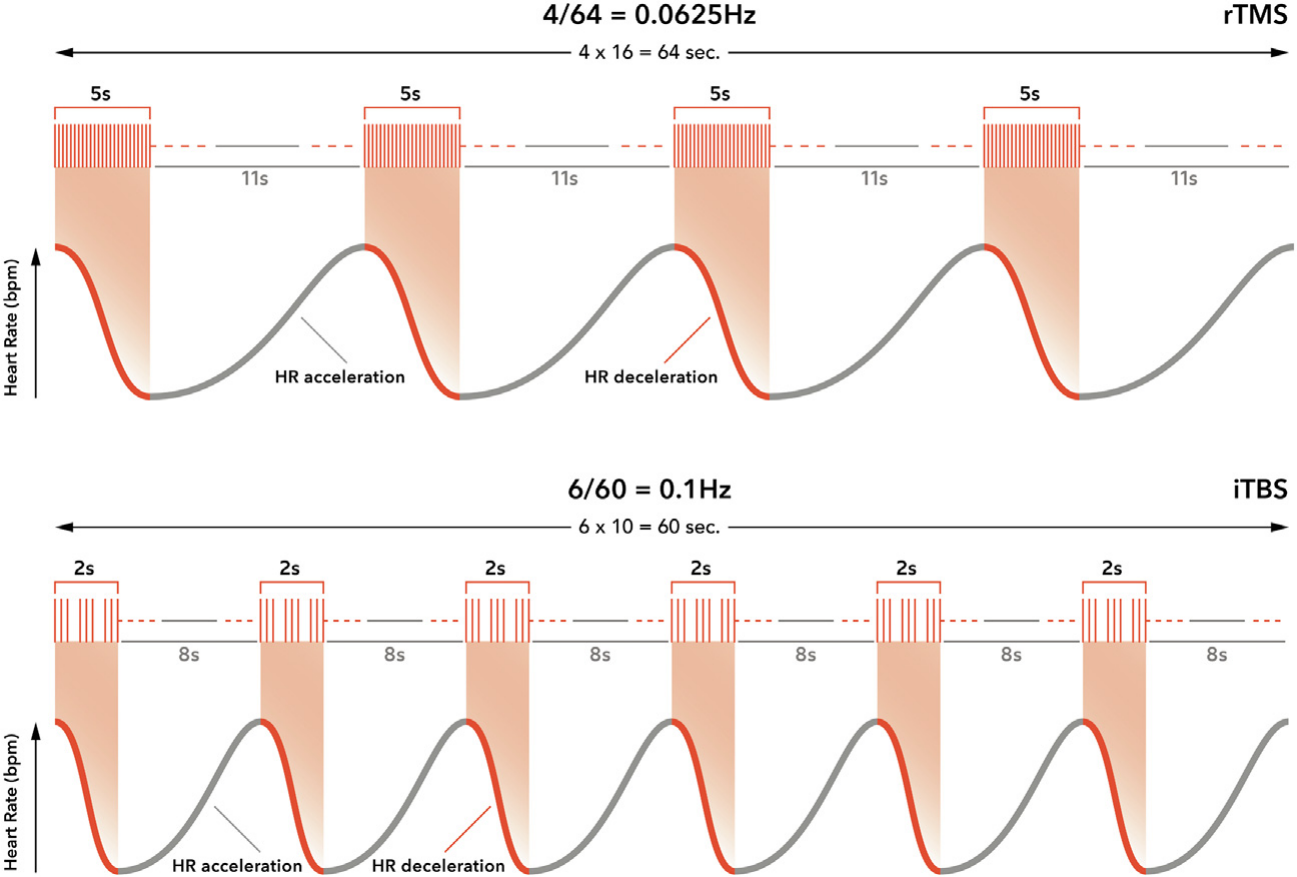
Repetitive transcranial magnetic stimulation (rTMS)–induced heart-brain coupling. This figure visualizes rTMS-induced entrainment of the interbeat interval as a function of rTMS cycle time. The cycle time of the 10-Hz Dash protocol comprises one rTMS train every 16 seconds (5 seconds stimulation on, 11 seconds stimulation off). This results in a specific entrainment frequency of 1/16 = 0.0625 Hz. For intermittent theta burst stimulation (iTBS), the cycle time is 10 seconds (2 seconds on and 8 seconds off), resulting in an entrainment frequency of 1/10 = 0.1 Hz. bpm, beats per minute; HR, heart rate.

We thus set out to test the above predictions for iTBS and the 10-Hz Dash protocol in 3 independent studies. Finally, as prior NCG-TMS studies have indicated that the frontal excitability threshold (FT) was not associated with the motor threshold (MT) (which is used to ‘dose’ prefrontal rTMS treatment) (8), we also tested if this method could be utilized to establish an FT. Two previous studies investigated the dose-response relationship between stimulation intensity of DLPFC-iTBS and clinical response. In healthy individuals, the largest neurophysiological changes were found at 75% MT, compared with 50% and 100% MT (14), and subthreshold iTBS resulted in a larger decrease of depressive symptoms than suprathreshold iTBS (15). These studies imply that higher TMS intensities could lead to attenuated effects, and research is needed to define the individual sweet spot of stimulation intensity.

## Methods and Materials

### Study 1: iTBS-Induced HBC

To test the specific entrainment of iTBS on the cardiac rhythm, we used data from 2 previous iTBS studies: baseline electrocardiogram (ECG) data collected at a Canadian health science center (author FV-R) from a double-blind placebo-controlled iTBS trial (CARTBIND; ClinicalTrials.gov Identifier: NCT02729792), reported in more detail in Blumberger *et al.* (16) and Iseger *et al.* (10) (study 1A), and ECG data from a double-blind randomized controlled trial performed at the Department of Psychiatry at Stanford University (Stanford Neuromodulation Therapy; ClinicalTrials.gov Identifier: NCT03068715), reported in more detail in Cole *et al.* (17) (study 1B). In summary, the methods comprised the following:

#### Participants

Fifteen (study 1A) and 22 (study 1B) patients with MDD were included, and all participants provided written informed consent. Exclusion criteria were 1) neurological/psychiatric disease, 2) age less than 18 years, and 3) standard exclusion criteria for rTMS, such as epilepsy.

#### iTBS Device and Protocol

iTBS-treatment localization was magnetic resonance imaging (MRI) based, and rTMS was applied with a MagPro X100 system (MagVenture) equipped with a B70 figure-of-eight coil (study 1A) and a double-sided Cool-B65 A/P coil (study 1B).

#### iTBS Device and Protocol: Study 1A

All subjects received 2 sessions of both sham and active iTBS. The active condition comprised stimulation at the DLPFC location with triplet 50-Hz bursts, repeated at 5 Hz; 2 seconds on and 8 seconds off; 600 pulses per session. A sham (internally shielded) coil without active electrical stimulation was positioned over the vertex during the sham condition. Subjects were randomized to one of the 2 treatment arms (arm A: sham-active, 54 minute pause, active-sham, or arm B: sham-sham, 54 minute pause, active-active). ECG was acquired simultaneous with iTBS stimulation using a Biopac MP 150 system (Biopac Systems Inc.) comprising modular hardware and AcqKnowledge software.

#### iTBS Device and Protocol: Study 1B

Subjects received either active or sham Stanford Neuromodulation Therapy: a high-dose iTBS protocol over the DLPFC, which consisted of 10 sessions (18,000 pulses) per day, on 5 consecutive days. Both sham and active iTBS were targeted at the left DLPFC. Participants and study staff were blinded to treatment assignments. All iTBS sessions utilized the same stimulation coil, with no indication of active or sham orientation. During sham, participants wore noise-canceling earphones connected to a sham noise generator to simulate the stimulation noise pattern. Additionally, lidocaine was applied to the stimulation site to reduce sensation. Spontaneous side effects were recorded daily. ECG was acquired simultaneously with iTBS using NCG-engage (neurocare), and only the first recording for each participant was used for analysis.

#### Coil Positioning and MT: Study 1A

The target location was specified by reverse coregistration from a stereotaxic coordinate on the standard Montreal Neurological Institute (MNI-152) template brain onto each individual anatomical MRI. MNI coordinates for left DLPFC were x −38, y +44, z +26, drawn from a study identifying this site as optimal based on clinical outcomes and resting-state functional connectivity (2). Stimulation was delivered at 120% MT.

#### Coil Positioning and MT: Study 1B

During active and sham stimulation, the coil was positioned over the DLPFC using MRI-guided neuronavigation. The stimulation intensity at 90% MT was adjusted for depth of the identified MRI target. For safety, stimulation intensity never exceeded 120% MT.

#### Analysis

**Figure 2:**
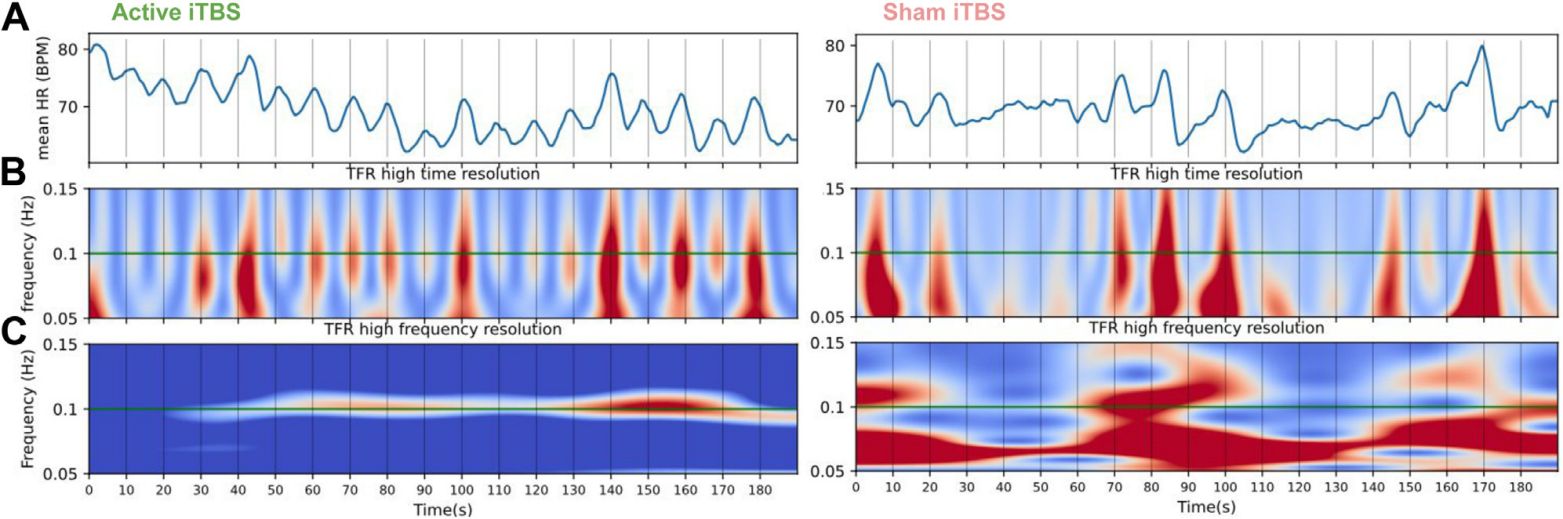
Heart-brain coupling report of active and sham intermittent theta burst stimulation (iTBS) condition. This overview visualizes the effect of one active and one sham iTBS session on heart rate (HR) **(A)** over 3-minutes time (x-axis), the 0.1-Hz high-time resolution **(B)**, and the 0.1-Hz high-frequency resolution **(C)**. The vertical lines represent the start of a stimulation train. Panel **(A)** visualizes that during stimulation, the heart rate consistently decreases and normalizes during the intertrain interval, in line with the hypothesized effects in Figure 1. This repetitive transcranial magnetic stimulation–induced cardiac rhythm of 0.1 Hz (green line) is also clearly visible as increased power (red) in the high-time and high-frequency plots. BPM, beats per minute; TFR, time-frequency representation.

From the ECG data of the first session, RR intervals (the time intervals between consecutive heartbeats) were determined using Kubios Premium [version 3.0.2, Kubios OY (18)], after which the data were analyzed using a custom-built analysis package (https://github.com/brainclinics/NCGTMS-2.0) in Python (Python Software Foundation). In short, HR was computed for each RR interval, and HR was interpolated for each ECG time point by a moving average of 5 consecutive HRs. Resulting HRs were convolved with a Hann window of 1.5 seconds (Figure 2A). Time-frequency representations were computed using the tfr_array_morlet function from the MNE-Python package (19) using a frequency range of 0.02 to 0.18 Hz in steps of 5 × 10^−4^ Hz, with 3 cycles yielding results in high-time resolution (Figure 2B) and 10 cycles for high-frequency resolution (Figure 2C). Before time-frequency representations analysis, data were padded with the first block of rTMS and rest period of data at the beginning and the last block of data at the end to allow for analysis at these low frequencies. To compute HBC, the mean power (μV^2^) at 0.1 Hz was computed for each block and averaged.

#### Analysis: Study 1A

For 3 subjects, insufficient ECG data were available, resulting in *n* = 12 included in the analysis. Researcher HvD—blinded to group assignment and stimulation details—processed the ECG data based on the above hypothesis of 0.1-Hz entrainment in RR signal and made predictions if ECG data belonged to sham or real stimulation within each subject. These predictions were examined after data lock by an unblinded researcher (MA). Next, a repeated measures analysis of variance (ANOVA) was conducted to test the effect of active and sham iTBS on the HBC marker (0.1 Hz).

#### Analysis: Study 1B

Three participants were excluded for low-quality ECG, resulting in *n* = 19. The ECG was analyzed by a blinded researcher (HvD), who used the same method as in study 1A to classify their active/sham status.

### Study 2: Dash (10-Hz) NCG-TMS

#### Participants

Ten healthy participants (7 female, age 25–46 [mean 34.0] years) were included in this study after providing written informed consent and meeting safety criteria for rTMS. Exclusion criteria were 1) neurological/psychiatric disease, 2) age less than 18 years, and 3) standard exclusion criteria for rTMS, such as epilepsy. This study was approved by the local ethics committee of Maastricht University.

#### rTMS Device and Protocol

**Figure 3:**
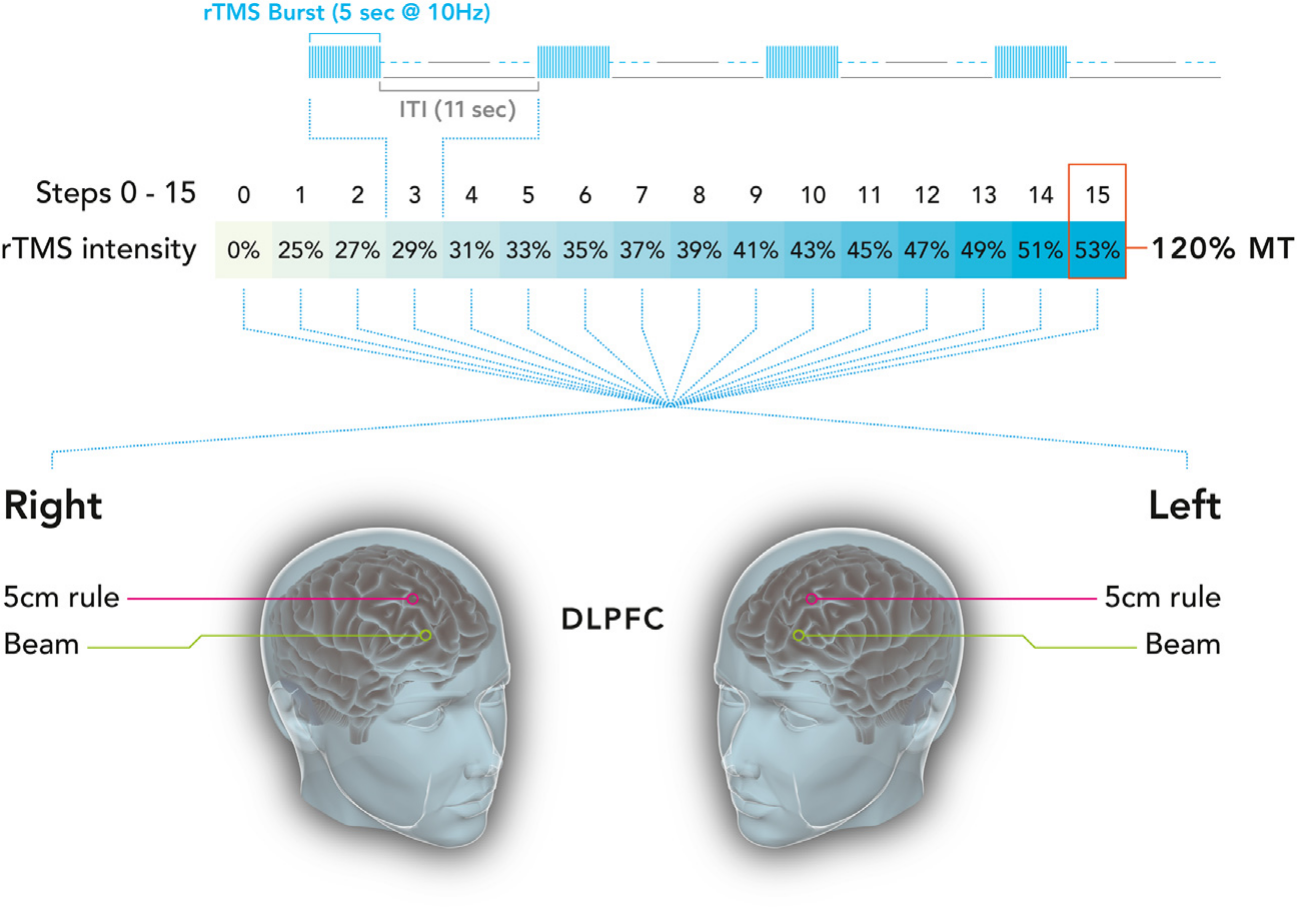
Visualization of the intensity sweep in study 2. This figure shows the intensity sweep that was applied in this pilot study 2, using 15 increasing intensities with 2% machine output steps. Step 15 corresponds to 120% motor threshold (MT). This repetitive transcranial magnetic stimulation (rTMS) protocol was applied on 4 sites: the Beam and 5CM locations on both hemispheres. 5CM location indicates the location 5 cm anterior to the scalp position for optimal activation of the first dorsal interosseus muscle in a parasagittal line. DLPFC, dorsolateral prefrontal cortex; ITI, intertrain interval.

rTMS was applied with either a DuoMag XT-100 system (Deymed Diagnostic) or a MagPro R20 system (MagVenture), both equipped with a focal figure-of-eight coil. Stimulation was applied in trains of 10 Hz for 5 seconds with an ITI of 11 seconds (Dash protocol). Subjects were presented with an intensity sweep of 15 stimulation trains from low to high intensities defined in 2% machine output steps, with 1 stimulation train (10 Hz for 5 seconds + 11 seconds ITI) per intensity, and with the 15th intensity matching 120% MT. This stimulation train was preceded by 16 seconds of no stimulation (total 256 seconds), and the starting intensity of machine output was thus defined as 28% machine output below 120% MT (Figure 3). This stimulation sequence was applied to 4 different locations (Beam and 5CM, left and right hemisphere; the starting location was randomized between subjects). During rTMS sessions, HR was simultaneously measured using an H10 Polar band (Polar Electro) connected through bluetooth with the ECG recorder app (version 1.4, Philipp Pöml).

#### Coil Positioning and MT

The rTMS coil was positioned over the Beam and 5CM location at an angle of 45° relative to the parasagittal plane (the coil handle pointing posteriorly), which seems to be the optimal angle to stimulate frontal areas (20) and which is used in most depression trials (21). If the distance between these two locations was smaller than 1 cm, the Beam and 5CM location on that hemisphere were taken as one location (*n* = 1). The 5CM location is defined as the location 5 cm anterior to the scalp position for optimal activation of the first dorsal interosseus muscle in a parasagittal line. Beam locations were defined using the Beam-F3 algorithm and software (22). MT intensity was defined as the lowest stimulation intensity that, in 4 trials, induced at least 2 visible twitches in the contralateral hand.

#### Analysis

**Figure 4:**
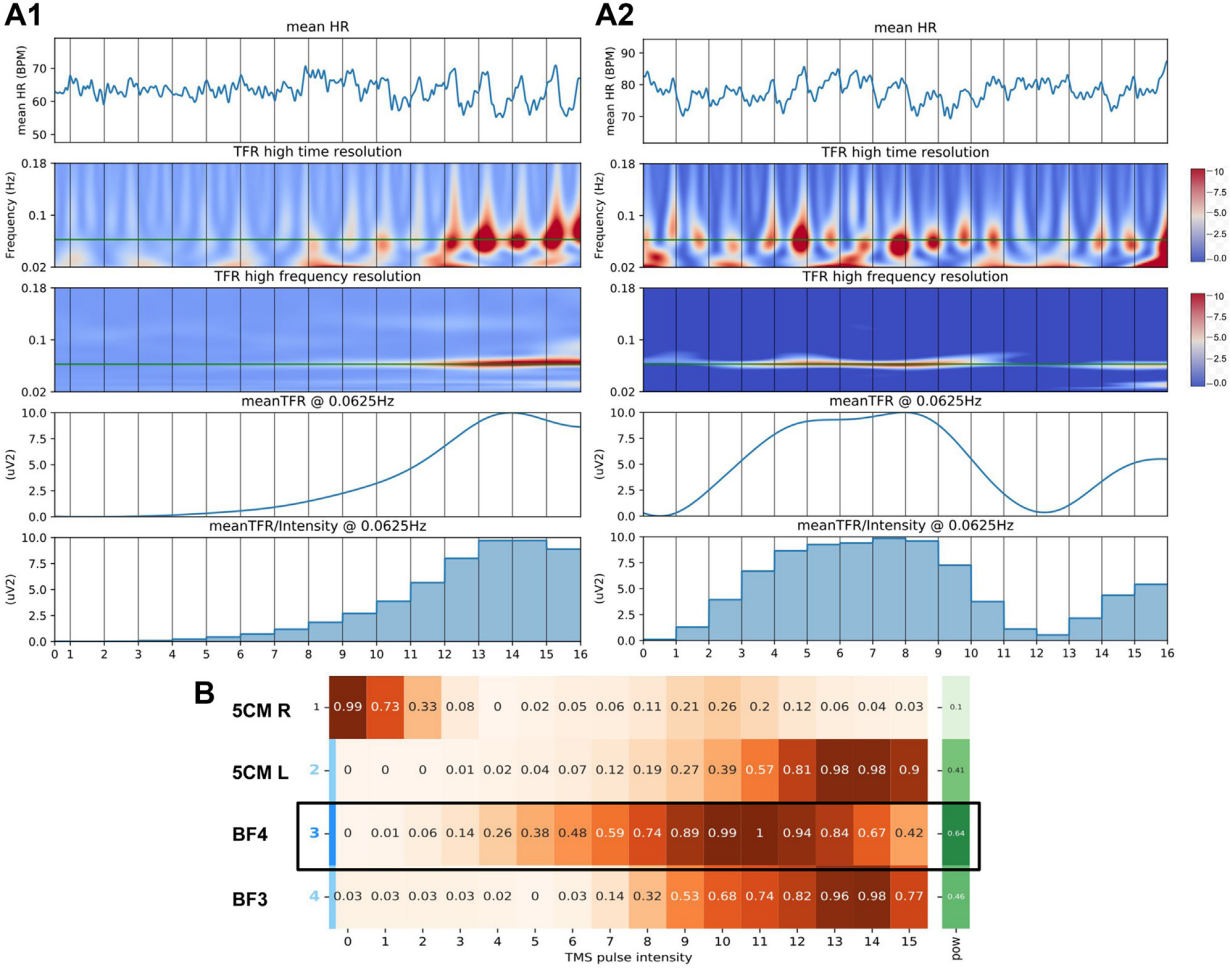
Heart-brain coupling (HBC) report example. This figure shows HBC power of 0.0625 Hz over time during the intensity sweep for one location in two different individuals (panels **A1** and **A2**). Steps 0 to 15 indicate the intensity sweep of increasing stimulator output, where step 0 is no stimulation. HBC is seen later in time for the individual in **A1** compared with the individual in **A2**, suggesting individual differences in low and high threshold response. Panel **(B)** shows the summary of HBC power of 0.0625 Hz during the intensity sweep for all stimulation locations for the individual in **A1**. Here, beam F4 location (BF4) would be the selected best location given the highest average oscillatory power at 0.0625 Hz (“pow”) at that location. 5CM, 5-cm rule; BPM, beats per minute; BF3, beam F3 location; HR, heart rate; L, left; R, right; TFR, time-frequency representation; TMS, transcranial magnetic stimulation.

Data were analyzed using the custom-built analysis package described above, now including the determination of the RR intervals. For this, ECG was bandpass filtered with a bidirectional fourth-order Butterworth filter between 5 and 49 Hz, after which signal.find_peaks [Scipy (23)] was used to detect R peaks. RR intervals were then corrected for ectopic beats, and subsequent analysis was performed as described above, in this case for 0.0625 Hz. HBC was quantified across the intensity sweep and 4 sites and visualized in a custom HBC marker report (Figure 4) (see the Supplement). Primary outcome measure was the HBC marker for the 10-Hz Dash protocol (0.0625 Hz). Using the HBC reports, the “best” location was determined for every subject based on the location with the highest average oscillatory power (μV^2^) at 0.0625 Hz (i.e., over all intensities) (see the Supplement). Data for all target locations and for the best location were subsequently analyzed in SPSS (version 28.0.1.0, IBM Corp.) with repeated measures ANOVA, using within-subject factors intensity (0–15) and location (BF3, BF4, and 5CM left and right).

## Results

### Study 1A

The blinded classifications on sham and active iTBS conditions from the ECG of each subject were highly accurate, with 10 of 12 predicted correctly by the blinded researcher (accuracy = 83%), suggesting successful unblinding of stimulation only by inspection of the ECG-based HBC marker.

Two examples of HBC output for a representative example of sham and real iTBS in the same subject are seen in Figure 2. This overview visualizes the effect of one 3-minute iTBS session on mean HR (Figure 2A) and 0.1-Hz HBC, visualized using a high-time resolution (Figure 2B) and high-frequency resolution (Figure 2C). Vertical lines represent the start of each stimulation train. During active iTBS, HR decelerates during every stimulation train (2 seconds) and normalizes afterward (8 seconds), until the next stimulation train, resulting in an rTMS-entrained HR oscillation at 0.1 Hz (horizontal green line), also visible as increased power (red) in the high-time and high-frequency plots. This is not seen during sham stimulation.

Repeated measures ANOVA yielded a main effect of stimulation (*F*_1,10_ = 16.318; *p* = .002, *d* = 1.37) and a main effect of time (*F*_1,10_ = 11.767, *p* = .006; *d* = 0.54), but no stimulation × time interaction (*p* = .318). Median and mean self-rated pain scores during active stimulation, on a scale from 1 (no pain) to 10 (intolerable pain), were higher (4.0 and 4.3, interquartile range = 2.7–6.0, SD = 2.0) than that during sham stimulation (1.0 and 1.1, interquartile range = 1.0–1.1, SD = 0.1).

### Study 1B

The generated HBC values predicted active/sham conditions with 89.5% accuracy. These results show that iTBS stimulation can be clearly distinguished from sham iTBS, based on the ECG-based HBC marker only, also when sham and active stimulation are targeted at the exact same location. Of measured spontaneous side effects, there was only a higher incidence of headache in the active iTBS group than the sham group (Fisher’s exact test, *p* < .06). Participants did not guess their treatment allocation beyond chance in both groups.

### Study 2

**Figure 5:**
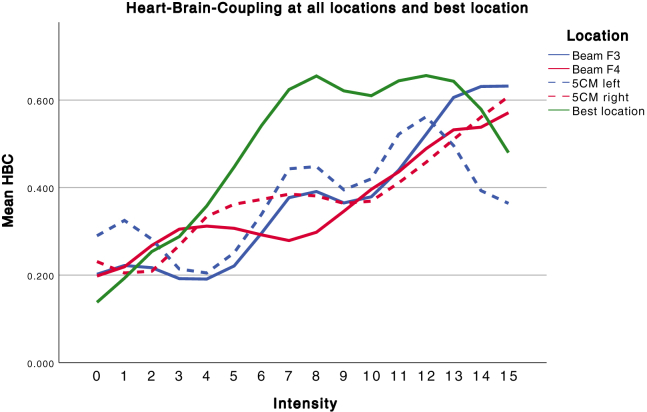
Dose-response effect of increasing stimulation intensities on heart-brain coupling (HBC). Dose-response effect of increasing intensities of machine output on the HBC marker in all locations (1–4) and the individual best location (5). The HBC effect for the best location has a higher maximum than the separate locations. The data in Figure 5 are derived from the individual HBC reports (see the Supplement), which were normalized to a 0–1 scale.

The repeated measures ANOVA on data of all locations showed a significant effect of intensity (*F*_15,135_ = 3.540, *p* < .001) on the HBC marker and no effect of location (*p* = .997) or location × intensity interaction (*p* = .955). The repeated measures ANOVA conducted on data for the best location per individual demonstrated a significant effect of intensity (*F*_15,135_ = 3.718, *p* < .001). The HBC peak for the best location, defined as the stimulation intensity at which the HBC reached the highest oscillatory power at 0.0625 Hz, reaches its maximum at intensity 8, with a large effect size (*d* = 2.40) (Figure 5). The data in Figure 5 were derived from the individual HBC reports (see the Supplement), which were normalized to a 0–1 scale. The Beam F3 and F4 were the individual best location for 2 subjects each, and the 5CM left and right for 3 subjects each. We investigated whether the order of stimulation had an impact on the HBC effect at different rTMS sites. No order effect was found, implying that HBC effects were independent of which site was stimulated first.

**Figure 6:**
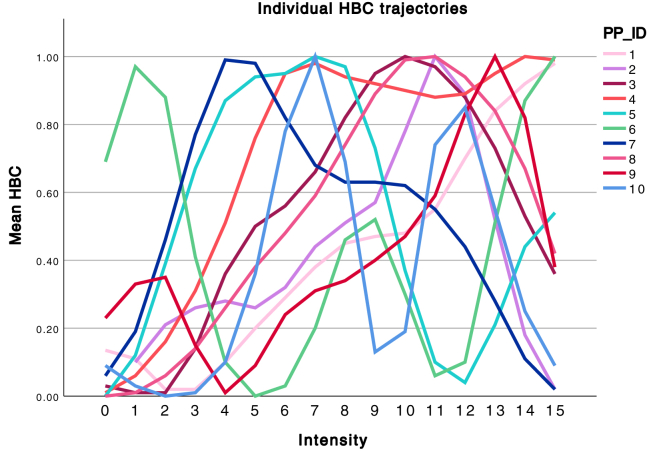
Individual trajectories of the heart-brain coupling (HBC) marker over time. Dose-response effect of increasing intensities of machine output on the HBC marker in all subjects. PP_ID, participant ID.

The results indicate a clear dose-response effect of rTMS-induced HBC, with no difference between sites at the group level. This is expected, as we are investigating a method to individualize rTMS targeting, based on the hypothesis that large interindividual differences exist. When the best site was chosen for each individual participant, a strong dose-response effect was observed. The dose-response relationship follows an inverted-U curve. More importantly, the data visualized in Figure 5 suggest large interindividual differences in FT, also confirmed by individual trajectories as visualized in Figures 4 and 6. The peak effect of stimulation on the HBC marker arises at different stimulation strengths for individual subjects (Figure 6), with the greatest difference between subject 10 (intensity 7) and 1 (intensity 15). These HBC peaks correspond with a relative %MT of 81% for subject 10 and 120% for subject 1. Importantly, for most of the individual subjects, the greatest effect arises sooner than step 15, which is currently the standard stimulation strength in clinical practice applied to frontal rTMS.

## Discussion

This study is the first to test and validate a novel NCG-TMS method utilizing HBC, which quantifies rTMS-induced entrainment of the heart interbeat interval as a function of TMS cycle time (Figure 1). The proposed model makes clear predictions about 2 commonly used clinical rTMS protocols used for the treatment of depression (24): iTBS with a cycle time of 10 seconds and 10-Hz Dash with a cycle time of 16 seconds. First, we validated this model for iTBS, in which the cycle time resulted in a 0.1-Hz entrainment, where we could successfully differentiate between active and sham iTBS with a large effect size and unblind 2 datasets with high accuracy. The effect of iTBS on HBC did not attenuate over time in study 1A [opposed to the effects of HR deceleration in the older NCG-TMS 1.0 (10)], which could be caused by neuroplastic effects of the first iTBS exposure. We further validated this finding for a second rTMS protocol (10-Hz Dash), where stimulation resulted in specific 0.0625-Hz HR entrainment.

In addition to target engagement results described above, study 2 also demonstrated clear dose-response effects on the HBC marker with large interindividual variability in FT. Prior studies have already demonstrated that NCG-TMS strength was not correlated to %MT but to % machine output, suggesting that %MT is a poor proxy for the FT (8). In our limited sample, the greatest difference in HBC peak was found between 2 subjects with a relative %MT of 81% and 120% (Figure 6). These different TMS-HBC distributions could reflect a combination of TMS intensity and possibly another factor, such as pain, target engagement, or individual differences in susceptibility to TMS. In larger samples, it is likely that larger differences will be found, necessitating studies to investigate this method further as an FT technique. The nonlinear and nonsigmoidal dose-response effects suggest possible overstimulation, which is in line with a recent case of TMS-induced syncope (25) and previous studies that imply that the stimulation intensity sweet spot neither under nor overdoses (14,15). Compared with those studies, we used more and smaller incremental steps in the current study and investigated HBC on an individual level, enabling the determination of an individual fine-grained dose-response curve.

Some potential limitations should be acknowledged. In all 3 studies, small sample sizes were included, decreasing the power of the studies. Studies 1A and 1B are hard to compare, as they differ in the number of TMS pulses (1A: 1200 pulses in 2 sessions; 1B: 90,000 pulses in 50 sessions) and the design (1A: within-subject design; 1B: between-subject design). In addition, in study 1B and study 2, subjective unpleasantness of stimulation was not measured; study 1A lacked a good control condition (vertex vs. DLPFC), and active condition was more painful than sham; and study 1B was not a within-subject design (participants could not compare sham and active condition directly). Therefore, distinctive scalp sensations might have contributed to differences between active and sham conditions. The sensory effects of TMS, such as the pain and discomfort associated with stimulation and its influence on HR and HBC, should be controlled for in future TMS studies by measuring pain and discomfort during stimulation and use that as a covariate or by conducting stimulation inside an MRI to demonstrate correlation between blood oxygen level–dependent changes and HR changes. This is important, as it is known that frontal TMS is especially uncomfortable (26) and can influence results such as reaction times in cognitive tasks (27). This was not sufficiently controlled for in the current study, necessitating future research to investigate the potential relevance of these results. Finally, it should be acknowledged that much is still unknown about the FT; hence, it is unclear whether the individualized FT has any associations or implications for clinical outcomes of rTMS treatment.

Despite the limitations, there are at least 3 good reasons to believe that the TMS protocol has direct effects on the frontal-vagal system and that nonspecific effects of rTMS, such as somatosensory stimulation, are unlikely to have affected the results:

1. In study 2, the HBC effect demonstrated clear site specificity. For nonspecific effects, a similar effect on the HR would have been expected for all 4 stimulated locations.
2. In addition, discomfort/pain at the stimulation site could affect HR, but mostly by sympathetic activation, and thus HR increases (28). Therefore, HR decelerations during rTMS stimulation can be interpreted as a specific effect on the frontal-vagal system.
3. Finally, in earlier work (29), rTMS was applied in anesthetized rats, and they found HR decelerations after active stimulation but not sham, emphasizing active signaling rather than just the effect of pain.

Our findings suggest that the use of rTMS-induced HBC might be a valuable method for rTMS target engagement of the frontal-vagal pathway. Given this novel approach deviates substantially from the earlier published NCG-TMS studies, we propose to refer to this method as NCG-TMS 2.0 or heart-brain coupling. In previous studies, it was shown that the effects of rTMS on HR are similar between healthy subjects and patients with MDD (5, 6, 7, 8, 9), which demonstrates the possible clinical implications of this novel method for target stratification in patients with MDD. In line with the suggestions by (9), this method could specifically be used to stratify patients with MDD between one of the two evidence-based stimulation clusters: the Beam and 5CM cluster. Large effectiveness studies have demonstrated comparable response (47%–58%) and remission (29%–37%) rates for these clusters (24,30); thus, NCG-TMS 2.0 could be used to effectively address interindividual differences between patients with MDD and stratify patients to their optimal rTMS target. This could enhance response and remission rates on an individual level, while adhering to validated rTMS protocols, as both targets are widely implemented in clinical practice.

Future studies should focus on replicating these findings in larger samples and investigate whether NCG-TMS 2.0 should be used for rTMS target stratification and for probing the FT patients in MDD to improve treatment outcomes. Further research should focus on optimization of other rTMS parameters as well, such as frequency of stimulation. In addition, the potential of NCG-TMS 2.0 could be investigated further for TMS in other (cognitive) applications. The current study will hopefully aid to individualize rTMS treatment and might have important implications for the field of stratified psychiatry. NCG-TMS may become for the prefrontal cortex, what the thumb twitch is for the motor system.

## Supplementary Material

Supplementary Material

Key Resource Table
