# Supplementary material for: Transcranial Magnetic Stimulation–Induced Heart-Brain Coupling: Implications for Site Selection and Frontal Thresholding—Preliminary Findings
Source: Biol Psychiatry Glob Open Sci. 2023 Jan 24;3(4):939–47. doi: 10.1016/j.bpsgos.2023.01.003 (PMC10593873; doi:10.1016/j.bpsgos.2023.01.003)

SUPPLEMENTARY INFORMATION

Transcranial Magnetic Stimulation-Induced Heart-Brain-Coupling: Implications for Site Selection and Frontal Thresholding: Preliminary Findings

Dijkstra *et al.*

Figure S1: Individual HBC report

This supplement shows an overview of the HBC-marker for one subject. Step 0-15 indicate the intensity-sweep of increasing stimulator output, in which step 0 is “no stimulation” and step 1 is the first stimulation round at the lowest intensity. Using the HBC reports, the ‘best’ location was determined for every subject, based on the location with the highest average oscillatory power (□V2) at 0.0625Hz (i.e. over all intensities). HBC=Heart-brain-coupling.

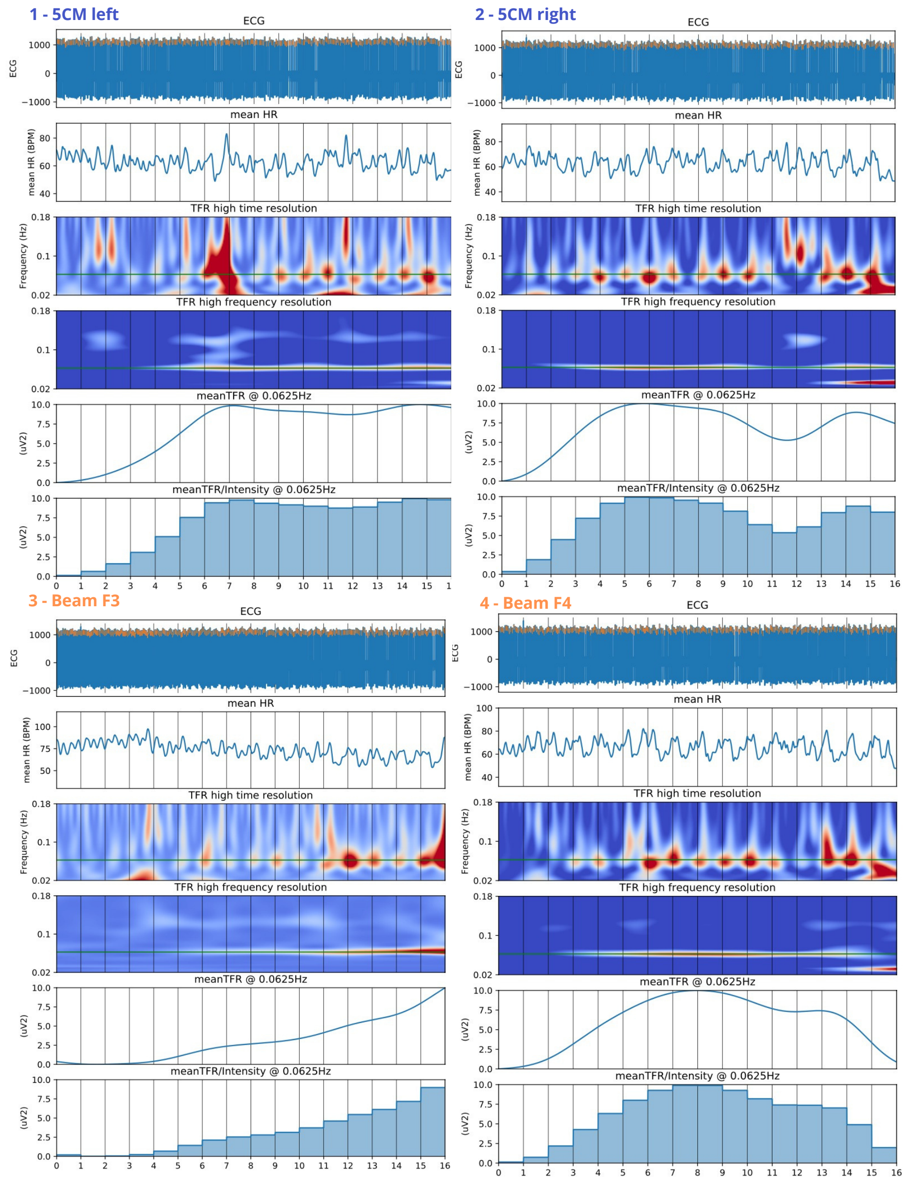

HBC marker score at all locations:

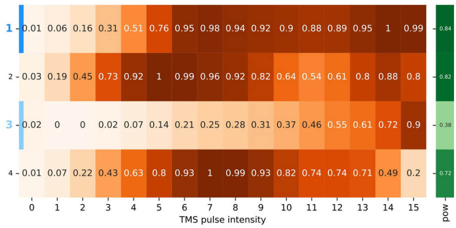

Supplement: Supplementary Material [file mmc1.pdf]
